# Supplementary material for: Ag2Se as a tougher alternative to n-type Bi2Te3 thermoelectrics
Source: Nat Commun. 2024 Aug 3;15:6580. doi: 10.1038/s41467-024-50898-6 (PMC11297924; doi:10.1038/s41467-024-50898-6)
Supplement: Supplementary file 1 — Supplementary Information [file 41467_2024_50898_MOESM1_ESM.pdf]

Supplementary Information

Ag_2Se as a tougher alternative to n-type Bi_2Te_3 thermoelectrics

Min Liu[1, #], Xinyue Zhang[1, #], Shuxian Zhang[1] and Yanzhong Pei[1, *]

[1] Interdisciplinary Materials Research Center, School of Materials Science and Engineering, Tongji Univ., 4800 Caoan Rd., Shanghai 201804, China.

[#]These authors contributed equally to this work, [*]Email: yanzhong@tongji.edu.cn

Table S1 Details for n-Ag_2Se/p-Bi_2Te_3 modules in this work and the commercial Bi_2Te_3 one.

Module	P type	N type	Pairs	Module size (mm^3)	Size of each leg (mm^3)
Commercial Bi_2Te_3 module	P-Bi_2Te_3 alloys, Model No. TEG1-712-0.14, Xiamen X-Meritan Technology Co., LTD.	N-Bi_2Te_3 alloys, Model No. TEG1-712-0.14, Xiamen X-Meritan Technology Co., LTD.	7	12×12×8.2	2×2×4
n-Ag_2Se/p-Bi_2Te_3 module 1	P-Bi_2Te_3 alloys, Model No. TEG1-712-0.14, Xiamen X-Meritan Technology Co., LTD.	This work, Ag_2Se with Ni electrode	7	12×12×8.2	2×2×4
n-Ag_2Se/p-Bi_2Te_3 module 2	P-Bi_2Te_3 alloys, Model No. TEG1-712-0.14, Xiamen X-Meritan Technology Co., LTD.	This work, Ag_2Se with Ag electrode	7	12×12×8.2	2×2×4

Table S2 Longitudinal (v_L) and transverse (v_S) sound velocities

Sample	Thickness (mm)	v_L (m/s)	v_S (m/s)
Ag_2Se (⊥)	0.78	1238.1	3250
Ag_2Se (//)	0.83	1220.6	3132.1
Average		1229.3	3191.0

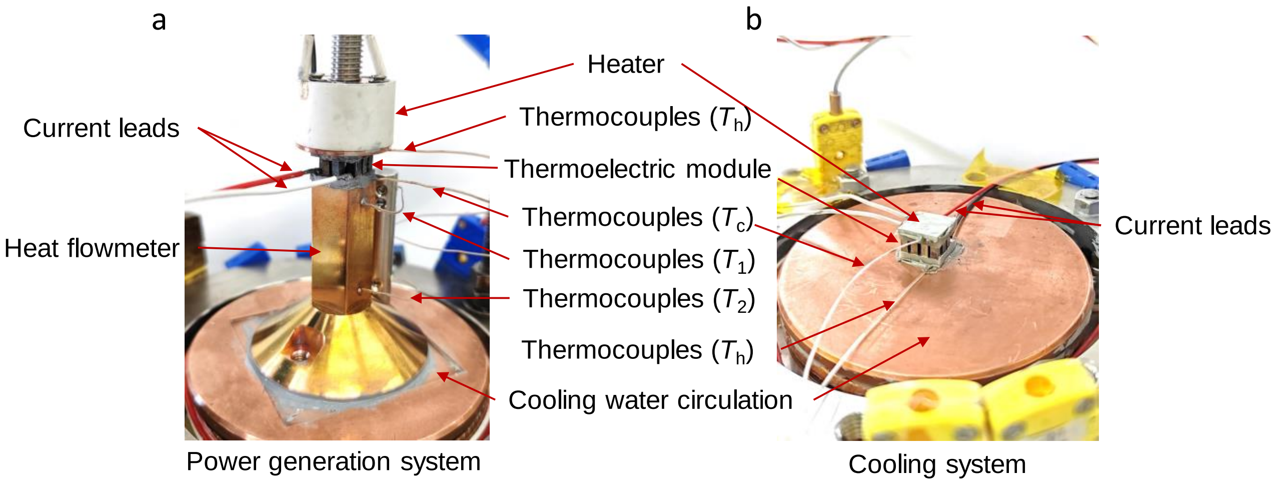

Fig. S1 Module measurement system. Photographs of (a) power generation and (b) cooling performance measurement setups for modules

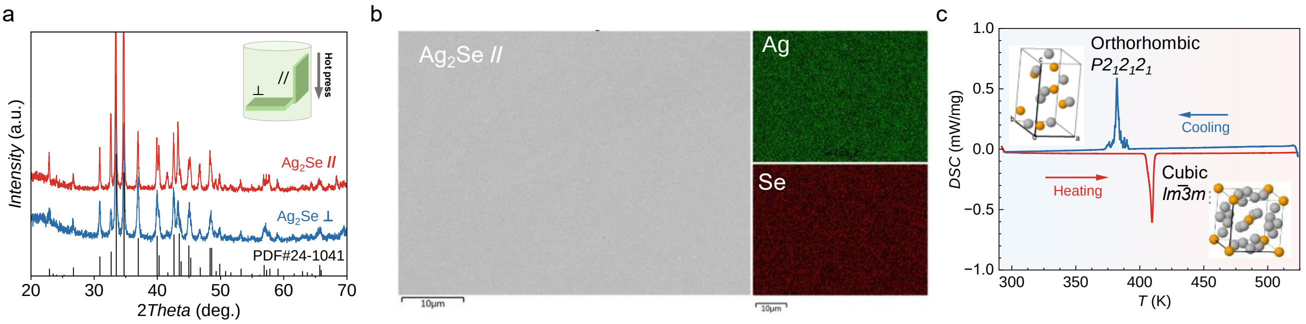

Fig. S2 Phase characterization of Ag_2Se. (a) XRD patterns of Ag_2Se pellets along directions perpendicular (Ag_2Se ⊥) and parallel (Ag_2Se //) to that of hot pressing, and (b) SEM images with corresponding EDS mapping for Ag_2Se //, (c) DSC curves of Ag_2Se during the heating and cooling process.

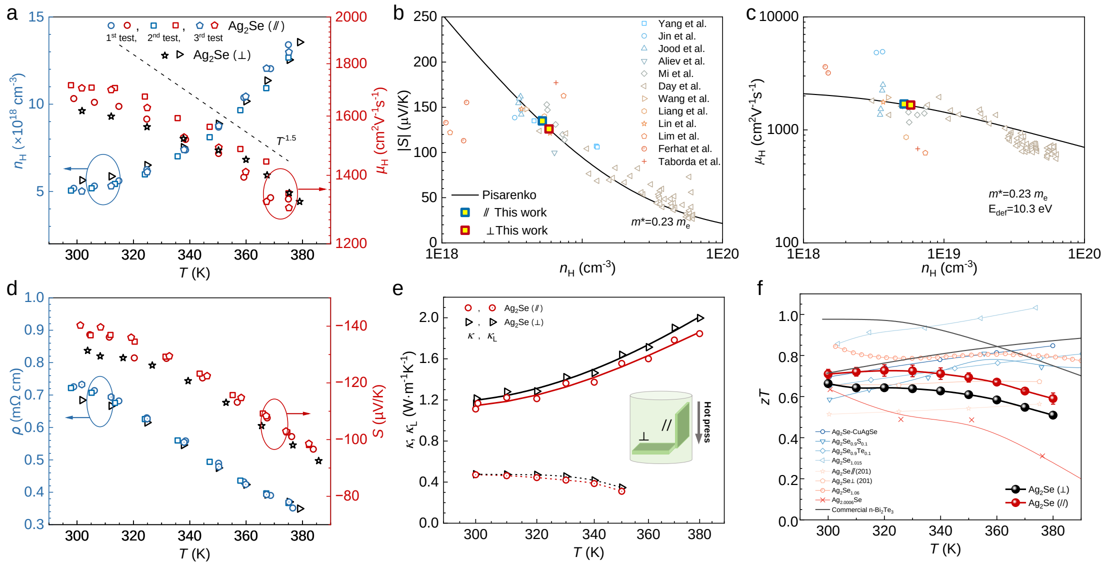

Fig. S3 Thermoelectric transport properties. Temperature dependent (a) Hall carrier concentration and Hall carrier mobility, (b) Seebeck coefficient *S*, electrical resistivity *ρ*, and (c) thermal conductivity *κ* and its lattice contribution κ_L; (d) Hall carrier concentration dependent Seebeck coefficient and (e) Hall mobility for Ag_2Se based on a single parabolic band model with acoustic scattering at 300 K, with literature results for a comparison[4-15]; (f) figure of merit zT at 300-380 K with a comparison to the literature results [8-12, 16-17].

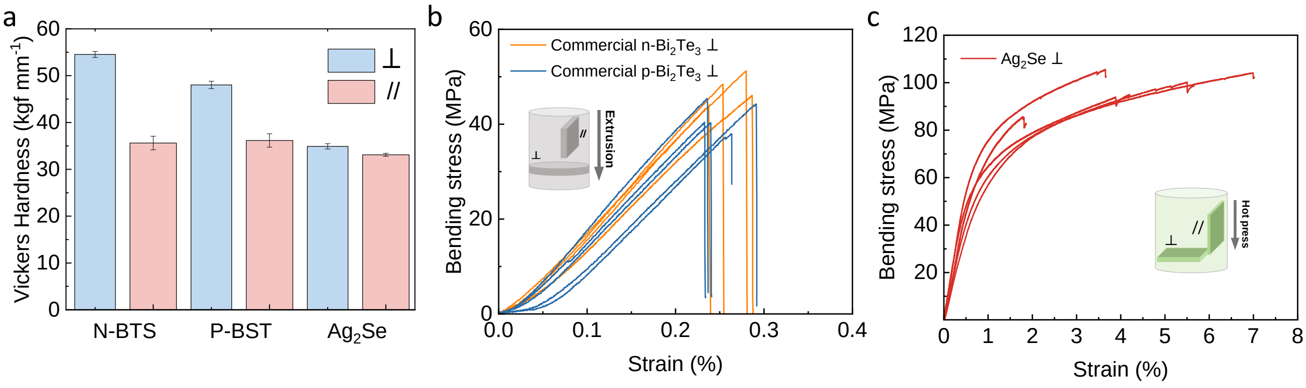

Fig. S4 Mechanical properties of commercial Bi_2Te_3. (a) Vickers hardness for hot-pressed Ag_2Se, commercial n-type and p-type Bi_2Te_3 by an extrusion technique, bending stress-strain curves of (b) commercial n-type and p-type Bi_2Te_3 and (c) Ag_2Se.

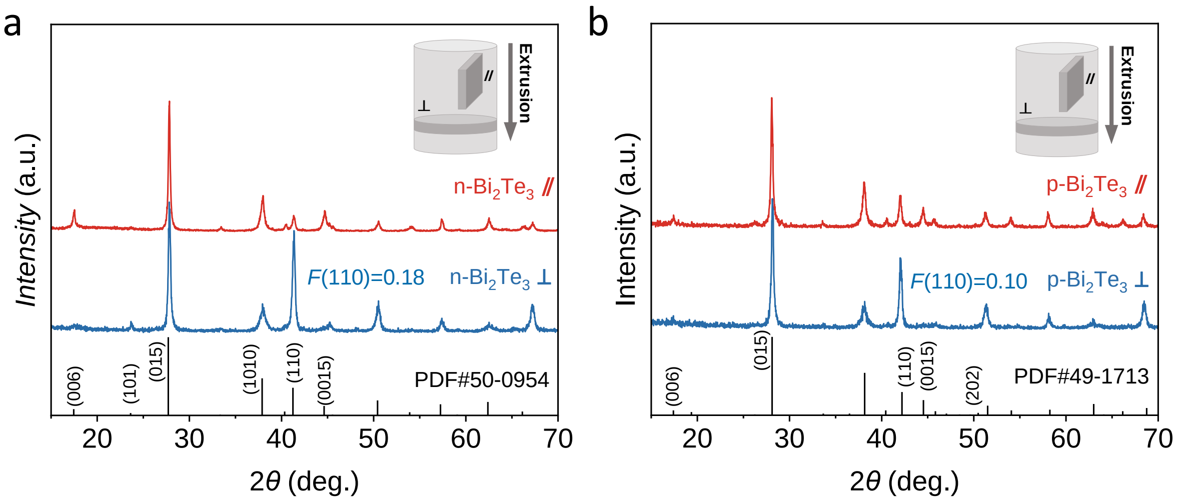

Fig. S5 XRD characterization. XRD patterns of commercial n-type (a) and p-type (b) Bi_2Te_3 by an extrusion technique along directions perpendicular and parallel to hot-pressing direction.

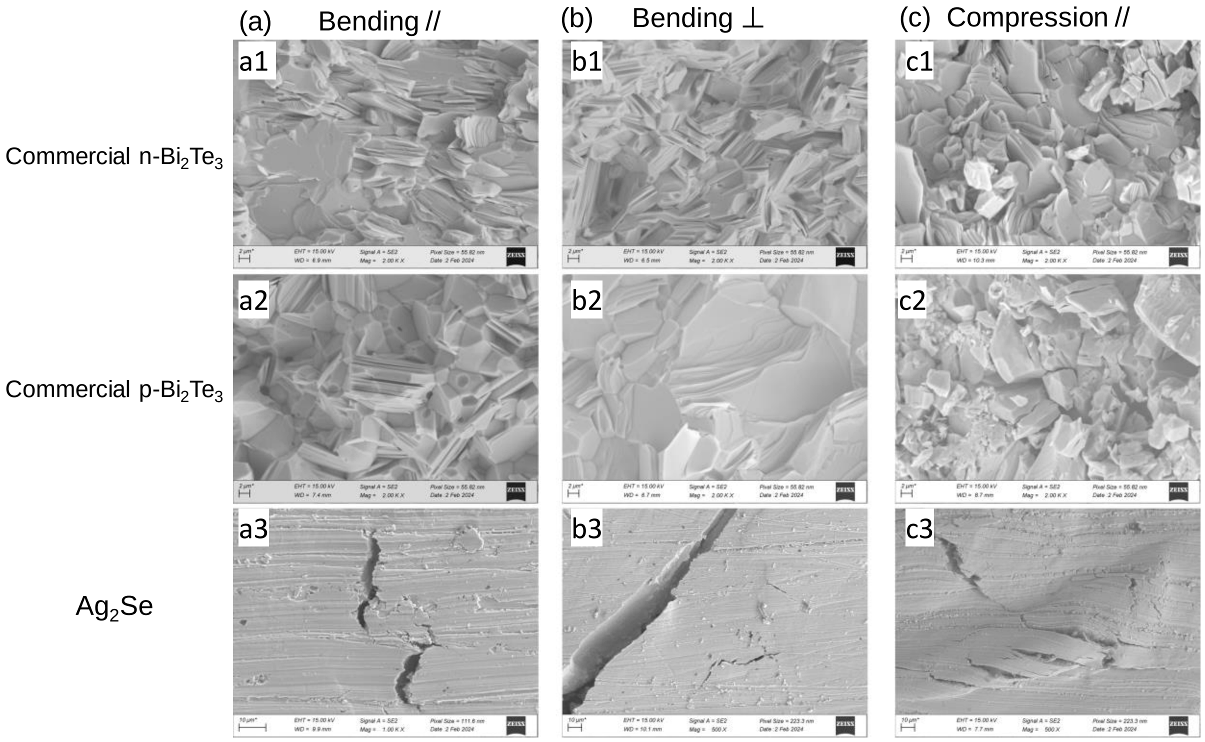

Fig. S6 Microstructure characterization. SEM images of the fracture surfaces of commercial n-type Bi_2Te_3, p-type Bi_2Te_3, and Ag_2Se after (a, b) bending (//, ⊥) and (c) compression (//) tests.

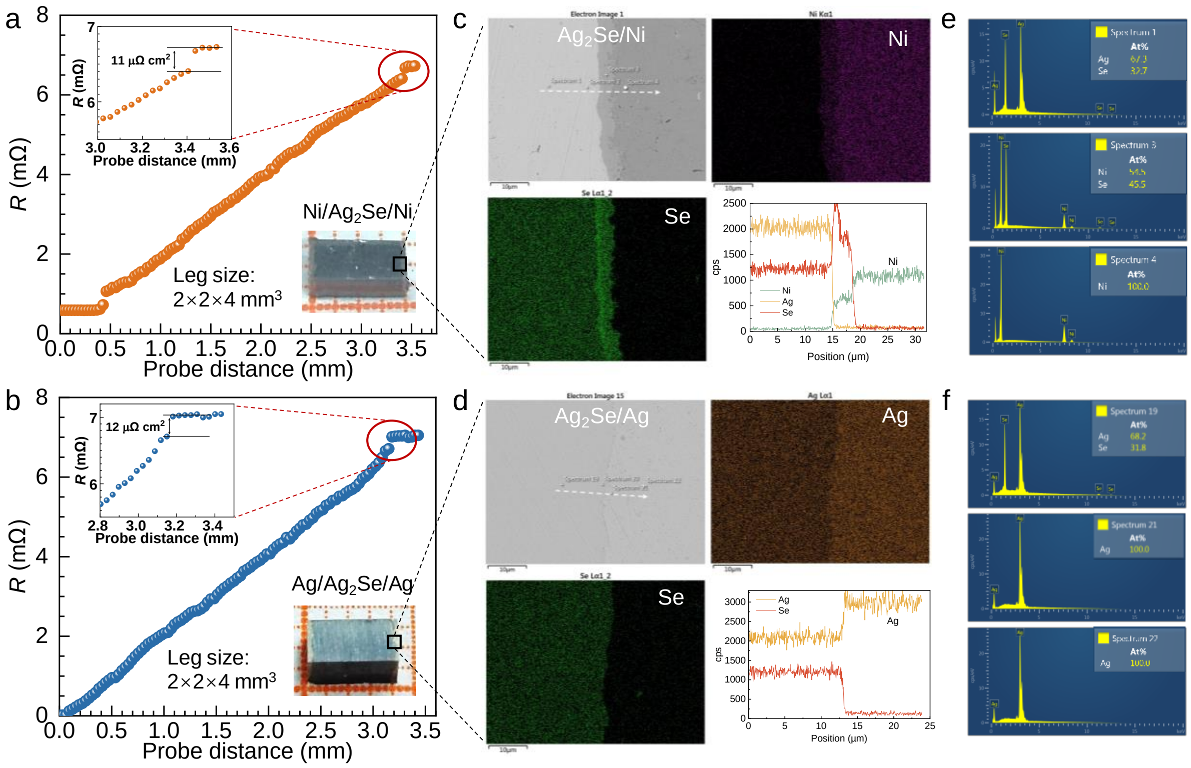

Fig. S7 Contact structure and resistance. Scanning resistance (*R*) across Ni/Ag_2Se/Ni (a) and Ag/Ag_2Se/Ag (b) junctions. SEM images, EDS mapping, EDS line scanning and elemental analysis for the Ag_2Se/Ni (c, e) and the Ag_2Se/Ag (d, f) joints.

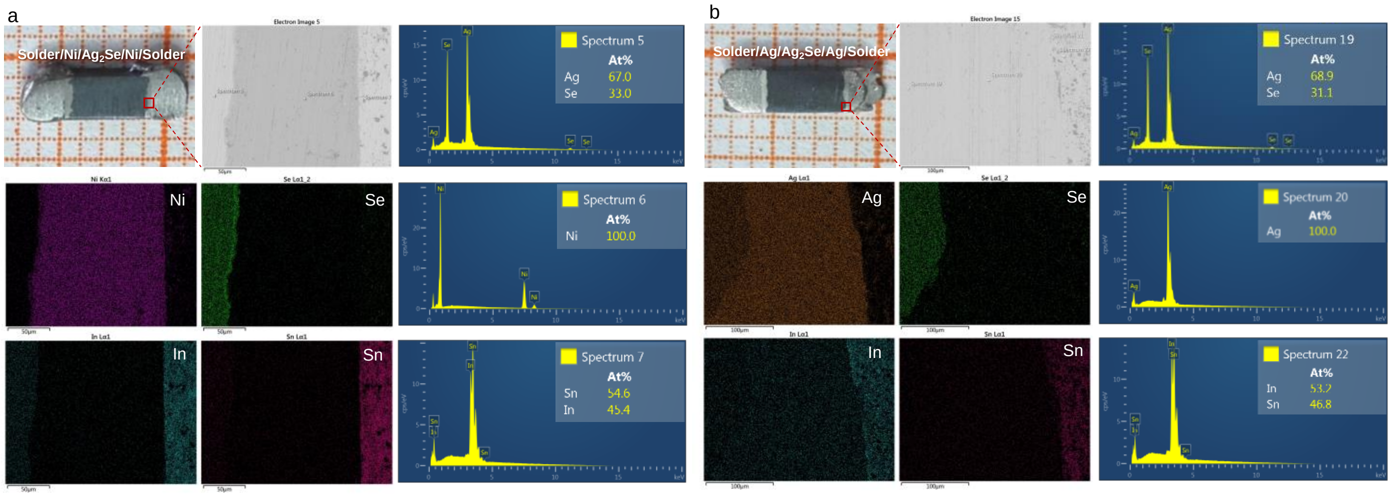

Fig. S8 Contact structure after welding. SEM images, EDS mapping and EDS elemental analysis for the Ag_2Se/Ni/solder (a) and the Ag_2Se/Ag/solder (b) joints.

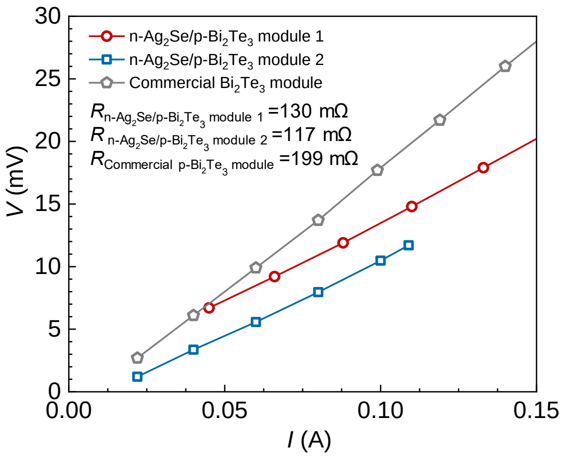

Fig. S9 Alternating current voltage as a function of current for both modules in this work and the commercial one.

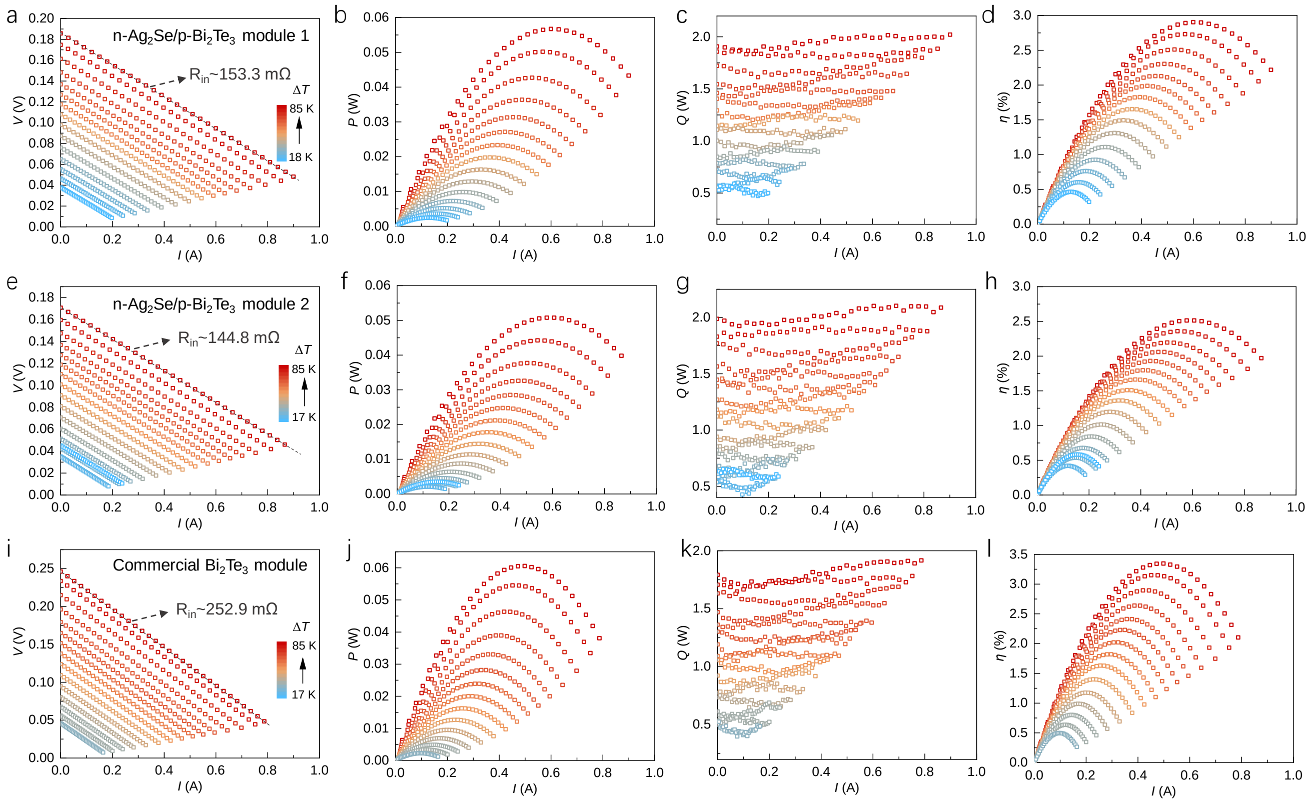

Fig. S10 Power generation performance versus current. (a, e, i) Output voltage V, (b, f, j) output power P (c, g, k), heat flow Q and (d, h, l) conversion efficiency (η) versus input current I at different temperature gradients (ΔT) for both modules here and the commercial one.

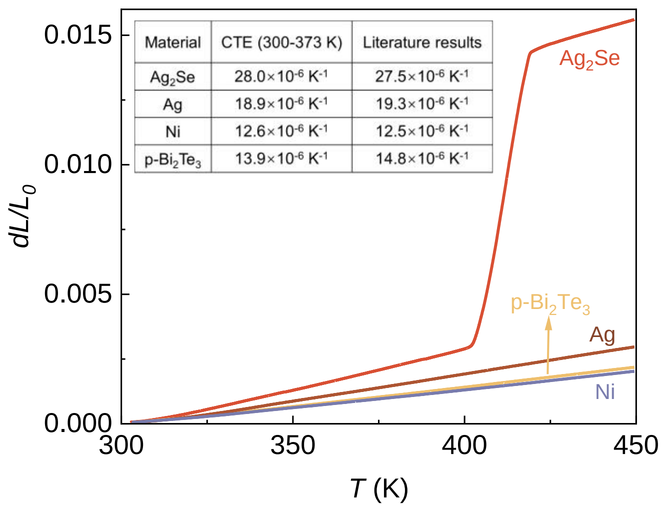

Fig. S11 Thermal expansion measurements. Temperature dependence of relative length variation (dL/L_0) for n-Ag_2Se, Ag, Ni and p-Bi_2Te_3 involved in our modules. The values on the dL/L_0 curves represent the linear coefficients of thermal expansion (CTE) in the specific temperature range. The inset shows the measured CTE at 300-373 K with comparison to literature results[5, 18-19].

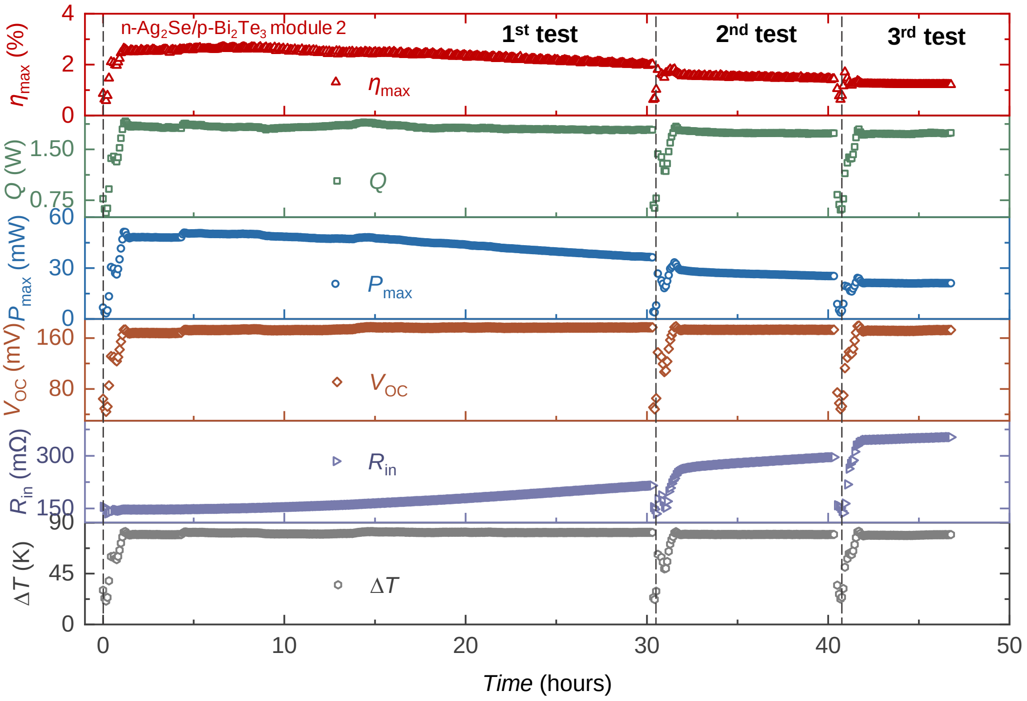

Fig. S12 Power generation duration of module 2. Maximum conversion efficiency η_{max}, heat flow Q, maximum output power P_{max}, open-circuit voltage V_{OC}, internal resistance R_{in} and ΔT of n-Ag_2Se/p-Bi_2Te_3 module 2 during duration measurements at ΔT of ~85 K.

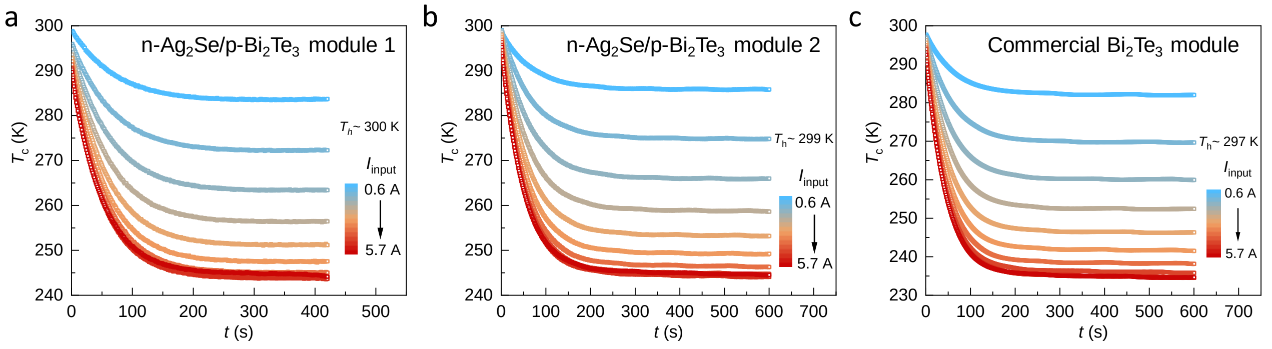

Fig. S13 Cooling performance. Cold-side temperature T_c as a function of time for (a, b) both modules and (c) the commercial one at different input current *I*.

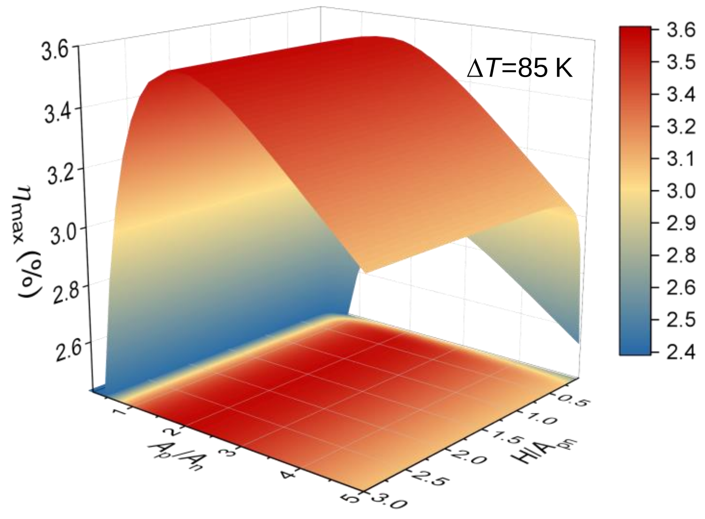

Fig. S14 Numerically simulated maximum efficiency (η_{max}) as a function of the p- to n-type ratio in legs' cross-sectional area (A_p/A_n) and the ratio of height to the total cross-sectional area of a pair of legs (H/A_{pn}) for the n-Ag_2Se/p-Bi_2Te_3 module. Over 90% of the theoretical optimal efficiency can be achieved when $1 \leq A_p/A_n \leq 4$ and $H/A_{pn} > 0.25$.

References

1. Lotgering, F. K., Topotactical reactions with ferrimagnetic oxides having hexagonal crystal structures—I. *Journal of Inorganic and Nuclear Chemistry* **1959,** *9* (2), 113-123.
2. Liu, M.; Zhang, X.; Tang, J.; Chen, Z.; Li, W.; Pei, Y., Screening metallic diffusion barriers for weldable thermoelectric devices. *Sci Bull (Beijing)* **2023,** *68* (21), 2536-2539.
3. Bu, Z.; Zhang, X.; Hu, Y.; Chen, Z.; Lin, S.; Li, W.; Xiao, C.; Pei, Y., A record thermoelectric efficiency in tellurium-free modules for low-grade waste heat recovery. *Nat Commun* **2022,** *13* (1), 237.
4. Yang, D.; Su, X.; Meng, F.; Wang, S.; Yan, Y.; Yang, J.; He, J.; Zhang, Q.; Uher, C.; Kanatzidis, M. G.; Tang, X., Facile room temperature solventless synthesis of high thermoelectric performance Ag_2Se via a dissociative adsorption reaction. *J Mater Chem A* **2017,** *5* (44), 23243-23251.
5. Jin, M.; Liang, J.; Qiu, P.; Huang, H.; Yue, Z.; Zhou, L.; Li, R.; Chen, L.; Shi, X., Investigation on Low-Temperature Thermoelectric Properties of Ag_2Se Polycrystal Fabricated by Using Zone-Melting Method. *The Journal of Physical Chemistry Letters* **2021,** *12* (34), 8246-8255.
6. Jood, P.; Ohta, M., Temperature-Dependent Structural Variation and Cu Substitution in Thermoelectric Silver Selenide. *ACS Applied Energy Materials* **2020,** *3* (3), 2160-2167.
7. Aliev, F. F.; Jafarov, M. B.; Eminova, V. I., Thermoelectric figure of merit of Ag_2Se with Ag and Se excess. *Semiconductors+* **2009,** *43* (8), 977-979.

8. Mi, W.; Qiu, P.; Zhang, T.; Lv, Y.; Shi, X.; Chen, L., Thermoelectric transport of Se-rich Ag_2Se in normal phases and phase transitions. *Appl Phys Lett* **2014,** *104* (13).

9. Day, T.; Drymiotis, F.; Zhang, T.; Rhodes, D.; Shi, X.; Chen, L.; Snyder, G. J., Evaluating the potential for high thermoelectric efficiency of silver selenide. *J Mater Chem C* **2013,** *1* (45), 7568-7573.

10. Wang, P.; Chen, J.-L.; Zhou, Q.; Liao, Y. T.; Peng, Y.; Liang, J. S.; Miao, L., Enhancing the thermoelectric performance of Ag_2Se by non-stoichiometric defects. *Appl Phys Lett* **2022,** *120* (19), 193902.

11. Liang, J.; Qiu, P.; Zhu, Y.; Huang, H.; Gao, Z.; Zhang, Z.; Shi, X.; Chen, L., Crystalline Structure-Dependent Mechanical and Thermoelectric Performance in $Ag_2Se_{1-x}S_x$ System. **2020,** *2020*.

12. Lin, S. Q.; Guo, L. L.; Wang, X. H.; Liu, Y.; Wu, Y. Y.; Li, R. B.; Shao, H. Z.; Jin, M., Revealing the promising near-room-temperature thermoelectric performance in Ag_2Se single crystals. *J Materiomics* **2023,** *9* (4), 754-761.

13. Lim, K. H.; Wong, K. W.; Liu, Y.; Zhang, Y.; Cadavid, D.; Cabot, A.; Ng, K. M., Critical role of nanoinclusions in silver selenide nanocomposites as a promising room temperature thermoelectric material. *J Mater Chem C* **2019,** *7* (9), 2646-2652.

14. Ferhat, M.; Nagao, J., Thermoelectric and transport properties of β-Ag_2Se compounds. *J Appl Phys* **2000,** *88* (2), 813-816.

15. Perez-Taborda, J. A.; Caballero-Calero, O.; Vera-Londono, L.; Briones, F.; Martin-Gonzalez, M., High Thermoelectric zT in n-Type Silver Selenide films at Room Temperature. **2018,** *8* (8), 1702024.

16. Chen, J.; Sun, Q.; Bao, D.; Tian, B.-Z.; Wang, Z.; Tang, J.; Zhou, D.; Yang, L.; Chen, Z.-G., Simultaneously enhanced strength and plasticity of Ag_2Se-based thermoelectric materials endowed by nano-twinned CuAgSe secondary phase. *Acta Mater* **2021,** *220*, 117335.

17. Chen, J.; Yuan, H.; Zhu, Y.-K.; Zheng, K.; Ge, Z.-H.; Tang, J.; Zhou, D.; Yang, L.; Chen, Z.-G., Ternary $Ag_2Se_{1-x}Te_x$: A Near-Room-Temperature Thermoelectric Material with a Potentially High Figure of Merit. *Inorg Chem* **2021,** *60* (18), 14165-14173.

18. Hirata, Y., Theoretical analyses of thermal shock and thermal expansion coefficients of metals and ceramics. *Ceram Int* **2015,** *41* (1), 1145-1153.

19. Shtern, Y. I.; Rogachev, M. S.; Bublik, V. T.; Tarasova, I. V.; Pozdniakov, A. V. In *The Results of Thermal Expansion Investigation for Effective Thermoelectric Materials*, 2019 IEEE Conference of Russian Young Researchers in Electrical and Electronic Engineering (EIConRus), 28-31 Jan. 2019; 2019; pp 1932-1936.
